# Psychological impact of the covid-19 pandemic on health care personnel working in COVID settings

**DOI:** 10.1192/j.eurpsy.2023.1727

**Published:** 2023-07-19

**Authors:** W. Ayed, D. Brahim, N. Mechergui, H. Ben Said, M. Mersni, S. Ernez, I. Youssef, N. Ladhari

**Affiliations:** Occupational pathology and fitness for work, Charles Nicolle Hospital, Tunis, Tunisia

## Abstract

**Introduction:**

COVID19 pandemic had a significant psychological impact on the population worldwide. However, health care workers have been the most exposed to psychological effects.

**Objectives:**

To determine the psychological impact of the covid19 pandemic on health care professionals (HCPs) who were working in the covid19 setting.

**Methods:**

Descriptive cross-sectional study carried out in May 2020, having interested the HCPs of the Charles Nicolle hospital who were working in the COVID19 settings .The data collection was carried out with a pre-established questionnaire .The visual analog scale of B. Chini was used to assess the level of work stress. This assessment was undertaken at three points in time: during the work, during the confinement period and post confinement .

**Results:**

Seventy five nurses participated to the study. The average age was 39.7±9.6 years. The sex ratio was 0.74. The average professional seniority was 11.6±8.14 years. During the confinement, a feeling of anxiety and apprehension of danger to others were reported by 96% of the participants. In addition, sleep disorders and irritability were noticed in 65% and 92% of cases respectively. At the end of the confinement period, 77% of the cases reported neuropsychological complaints: feelings of anxiety (57%), mood disorders (49%), a sleep disorders (32%) and concentration disorders (20%). The average level of stress was evaluated at 7.54 during the work, 7.36 during confinement and 5.28 after confinement. Faced with this psychological suffering, 88% of the cases noted the absence of psychological support or assistance.

**Conclusions:**

Psychological support and early screening in psychiatry and occupational medicine are necessary to prevent any deterioration in their mental health.

**Disclosure of Interest:**

None Declared

